# Comparative finite element analysis of fixation strategies for sports-related tibial tuberosity avulsion fractures in adolescents: biomechanical evidence for optimized clinical decision-making

**DOI:** 10.3389/fspor.2025.1730221

**Published:** 2025-12-02

**Authors:** Xiaoming Wang, Hengheng Zhang, Zhe Tuo, Siwen Liu, Hailiang Meng, Xing Tong, Ming Yang, Bing Wang

**Affiliations:** General Division, Pediatric Orthopedic Hospital, Honghui Hospital, Xi’an Jiaotong University, Xi'an, China

**Keywords:** tibial tuberosity avulsion fracture, finite element analysis, screwfixation, kirschner wire, biomechanics, adolescent

## Abstract

**Objective:**

This finite element study aimed to biomechanically compare screw fixation vs. K-wire fixation for adolescent tibial tubercle avulsion fractures (TTAF), investigating the differential biomechanical outcomes and physeal insult associated with each fixation method.

**Methods:**

A finite element model of an adolescent tibia with a Modified Ogden type III fracture was developed. Three fixation types—(A) screws, (B) two non-crossing K-wires, and (C) four crossing K-wires—were tested both as standalone constructs and in their tension band-augmented counterparts. Simulations applied a 1,654 N tensile load to simulate patellar tendon force, with outcomes assessed by fragment displacement, bone and epiphyseal von Mises stress.

**Results:**

Screw fixation (Type C3) demonstrated the highest stability, with fragment displacement of 1.97 mm and the lowest bone stress (295.79 MPa). K-wire constructs showed greater displacement and stress. The addition of a third fixator provided minimal stability improvement compared to two. Tension bands increased stability in K-wire models but raised bone stress to approximately 1,100 MPa and increased physeal stress.

**Conclusion:**

Biomechanical analysis of TTAF reveals that screw fixation provides enhanced stability and reduced bone stress compared to K-wire fixation. Furthermore, the stability achieved with dual screws is comparable to that of triple screws, implying that adding more implants offers a limited advantage in fracture fixation stability. Considering the greatest physeal insult, particularly in skeletally immature individuals with retained growth potential, tension band wiring should be used sparingly. Based on these results, screw fixation is recommended as the optimal approach for adolescent TTAF.

## 1 Introduction

Although once considered uncommon, the incidence of Tibial Tuberosity Avulsion Fractures (TTAF) has risen in recent years, a trend attributed to increasing adolescent sports participation and evolving diagnostic practices (1–3). These injuries most frequently affect adolescents between 13 and 17 years of age and exhibit a strong male predominance, consistent with other sports-related injuries (4–7). TTAF account for approximately 0.4%–2.7% of pediatric fractures and less than 1% of all epiphyseal injuries (8–10). The typical mechanism involves a forceful quadriceps contraction against resistance, often during jumping activities such as in basketball or football, or during a sudden deceleration while running.

Since the initial classification system was introduced in 1976, several modified classifications have been developed to better correlate fracture patterns with treatment strategies (10, 11). Despite these efforts, standardized surgical guidelines remain elusive, leading to significant variability in the management of these fractures in children (12, 13). A key area of uncertainty involves how combined fixation constructs (such as K-wires and tension band screws) affect the fragile epiphysis, and how to reduce associated complication risks.

The choice of fixation method is a primary determinant of complications in TTAF management. Proposed techniques include cannulated screws, K-wires, and suture tension bands. The use of plate constructs is typically contraindicated in the pediatric population due to the presence of an unclosed epiphysis. Reported late complications are relatively common and include premature physeal closure, knee stiffness, malunion, nonunion, and re-fracture (14). In adolescents with remaining growth potential, the potential for iatrogenic injury to the epiphysis demands particular caution. Nevertheless, a clear understanding of the biomechanical distinctions between various fixation methods is still lacking. While two parallel horizontal screws represent a commonly used and straightforward technique for TTAF fixation, no prior mechanical studies have comprehensively compared this configuration to alternatives. The internal mechanical environment—including the stress distribution within the bone fragment and the contact forces at the fracture site—remains uncharacterized.

Beyond overall construct stability, the internal mechanical state of the bone fragment following fixation—specifically, the stress concentrations and contact forces after static equilibrium—is poorly understood. Furthermore, complications often arise from fractures of the bone fragment itself, particularly in the region surrounding the screw holes. Stress risers in these areas are critical, as elevated bone stress is a potential precursor to mechanical failure. Quantifying these stresses experimentally is challenging due to the impracticality of placing sensors within the bone without altering its mechanical properties. In contrast, the finite element (FE) method offers a powerful numerical approach to non-destructively calculate internal stress and strain distributions. Therefore, this study utilized the finite element method to investigate the influence of the bone-tibia gap and different screw configurations on TTAF fixation stability, contact forces at the fracture interface, and bone von Mises stress.

## 2 Methods

A finite element model was developed to simulate adolescent Tibial Tubercle Osteotomy Fixation (TTAF) and evaluate the biomechanical performance of various fixation methods. The model incorporates a tibia with a simulated TTAF fixation, treating the fracture with either hollow screws or Kirschner wires in conjunction with tension bands. Fixation stability was assessed by comparing the displacement of bone fragments in different bone-internal fixation constructs. Furthermore, we analyzed the maximum intraosseous von Mises stress, contact forces at the fracture interface, and stress distribution within the physis to evaluate the potential risk of physeal injury. All simulations were conducted under linear static loading conditions. The model assumes all materials are homogeneous and isotropic. Nonlinear surface contact was defined to simulate interactions between all components, including the fracture surfaces and implant-bone interfaces.

The study was approved by the institutional review board, and written informed consent was obtained from the patient's guardian for the anonymous use of the imaging data.

### 2.1 TTAF model construction

The geometry for the knee joint model was derived from computed tomography (CT) scans of a 12-year-old male patient (height: 155 cm, weight: 45 kg) who underwent imaging following a traumatic tibial fracture. The CT (Philips, Amsterdam, The Netherlands) scan was performed with the following parameters: 120 kV, 30 mA, and a slice thickness of 1.0 mm, covering the region from the supracondylar femur to the proximal tibia, which yielded a total of 384 axial slices with a 512 × 512 matrix. Radiological evaluation and detailed medical history confirmed the absence of other musculoskeletal diseases. A fracture model was then constructed based on the proximal tibia.

Reconstruct the initial 3D model from CT data using Mimics 21.0 (Materialise NV, Belgium). Cortical bone segmentation was performed using a grayscale threshold applied to the CT images. The complex curvature of the physis was carefully modeled by delineating characteristic sclerotic margins observed on CT, which demarcate the interface between the epiphysis and metaphysis. Subsequently, the initial 3D model was imported into Geomagic Wrap 2021 [(3D Systems, Rock Hill, USA)] for geometric refinement. A high-fidelity surface model was achieved through editing and smoothing operations to process the initially rough surfaces. Given the ill-defined interface between cortical and cancellous bone in the proximal tibia on CT, the cancellous bone region was established by uniformly contracting the inner cortical surface by 2 mm (15).

### 2.2 Construction of bone-internal fixation assembly model

All implants and the final assembly model were constructed using SOLIDWORKS 2023, a parametric solid modeling software (Dassault Systems SolidWorks Corp, Waltham, USA). Based on the previously established fracture geometry, we developed a finite element model incorporating screw and Kirschner wire (K-wire) fixation to compare the biomechanical stability of different fixation constructs.

**Figure 1:**
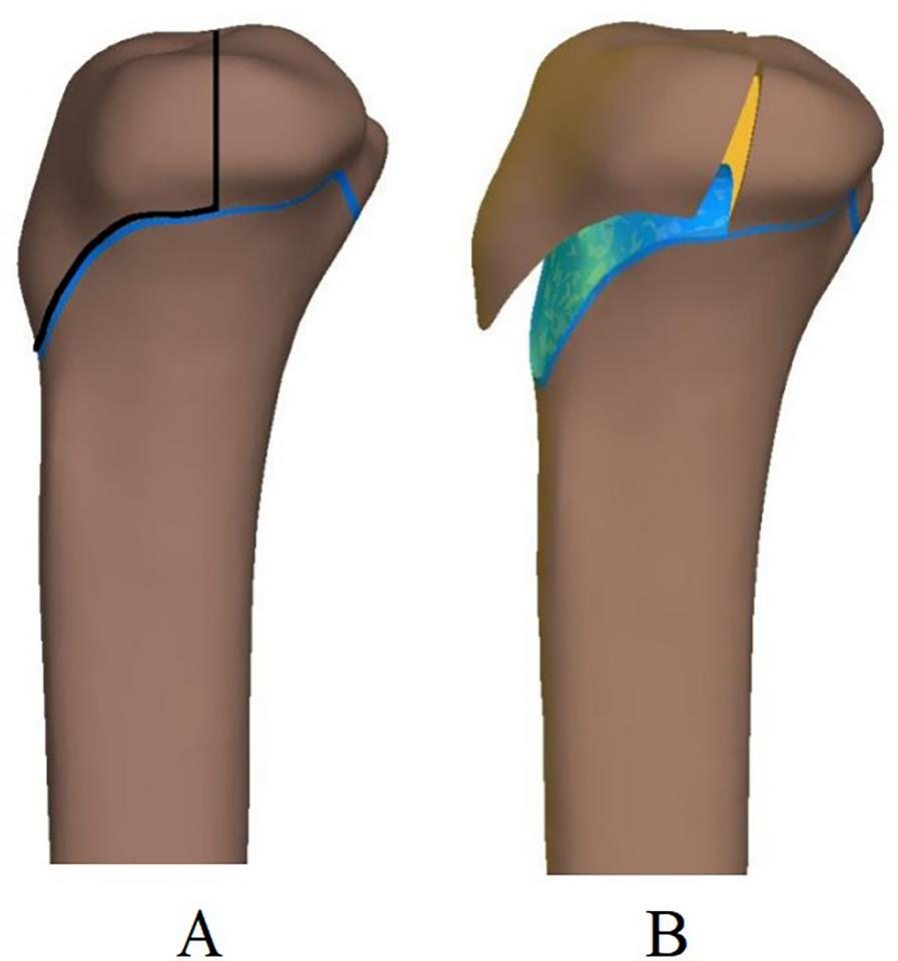
Bone model and modified ogden III fracture model. **(A)** Regional tibial model incorporating the epiphysis. **(B)** The fracture line originates at the tibial tuberosity and extends into the articular surface.

The fracture model simulated a modified Ogden Type III fracture, a representative pattern encompassing features of Type I to Type II tibial tuberosity avulsion fractures. The avulsed fragment involved epiphyseal detachment and intra-articular involvement, with the fracture line extending anteroposteriorly and superiorly to the articular surface (Figure 1).

**Figure 2:**
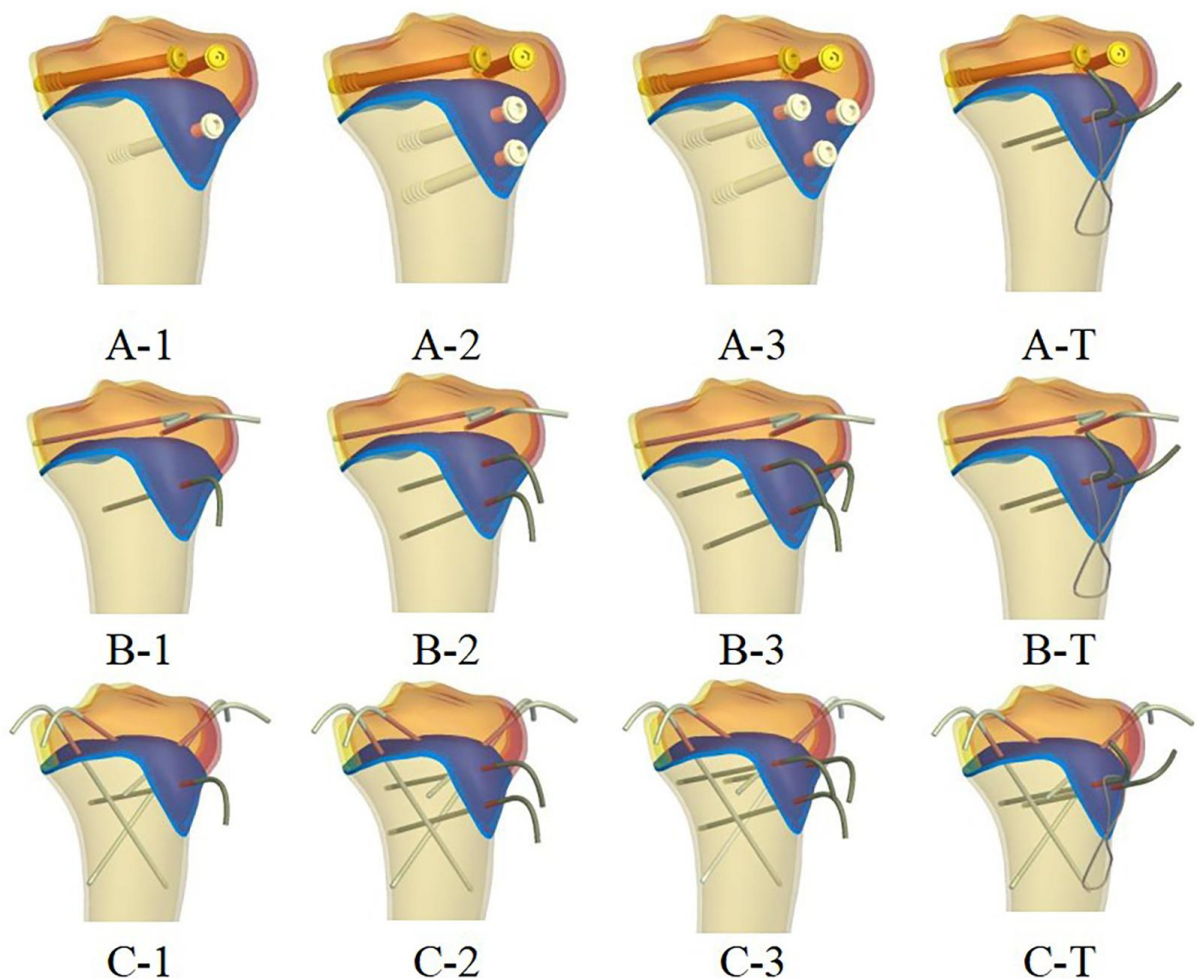
Fracture-internal fixation assembly.

The designed fixture was implanted into the bone model using SOLIDWORKS 2023's Boolean operations, thereby eliminating any volumetric overlap between the implant and the bone. Subsequently, the fractured fragments were fixated using the following implant configurations: 4.5 mm cortical screws, 2.0 mm K-wires, and 1.0 mm steel tension bands (fabricated from Titanium-6Al-4V alloy), thereby creating the fracture construct fixation model (Figure 2). Fracture reduction was idealized, with no gaps present at the fracture surfaces.

Three distinct fixation methods were modeled, with variations within each category, resulting in a total of four specific configurations:

Type A: Defined as standard screw fixation.
Type B: Defined as K-wire fixation, utilizing two K-wires that did not traverse the epiphyseal plate.
Type C: Also defined as K-wire fixation, but employing four K-wires configured to intersect and cross the epiphyseal fragment.

For each primary fixation method (A, B, and C), the tibial tuberosity fragment was fixed with one to three screws or K-wires, respectively, positioned in an oblique posteroinferior direction. Additionally, a tension band fixation model variant was created for each primary type, resulting in three supplementary models designated as A-T, B-T, and C-T.

### 2.3 Boundary conditions and material properties

**Figure 3:**
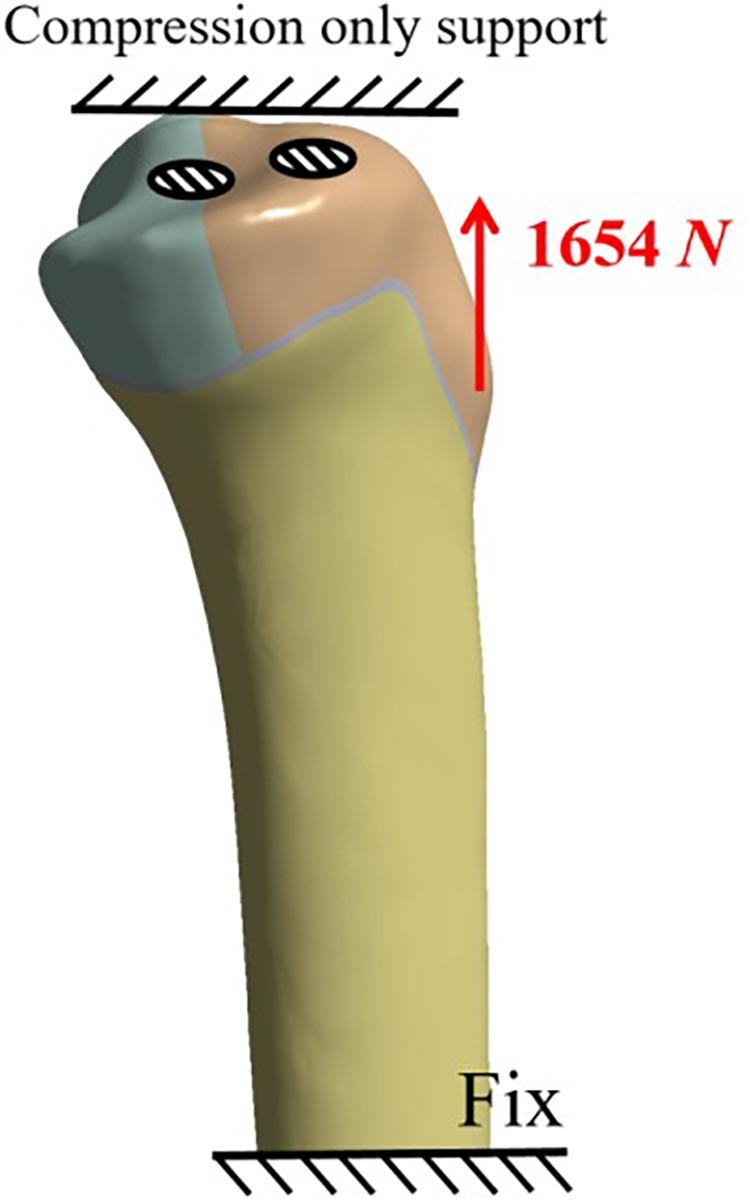
Schematic diagram of model load application.

The boundary conditions and applied loads were defined to simulate a worst-case scenario under extreme physiological load. A prior biomechanical study by Davis et al. (16) on fresh-frozen cadivers reported a failure load of 1,654 N for tibial tuberosity osteotomy fixed with two 4.5 mm cortical screws. Accordingly, this failure load was adopted in our FE analysis to model an extreme loading condition. To simulate the tension from the extensor mechanism, a 1,654 N tensile force was applied to the patellar ligament attachment site on the tibial tuberosity fragment (Figure 3). The direction of this force vector was determined based on the anatomical orientation derived from the CT data.

For boundary constraints, the entire superior surface of the tibia was assigned a compression-only support. This condition simulates the resistance provided by the distal femoral condyles, allowing the surface to transmit compressive normal forces but not tensile stresses. The distal end of the tibia was fixed in all degrees of freedom to prevent any rigid body motion of the model during the simulation.

### 2.4 Contact interactions and material properties

The contact interactions between the various model components were carefully defined to replicate physiological and surgical conditions. A frictional (nonlinear) contact formulation was applied to two critical interfaces (0.45): the surface between the fracture fragment and the fracture interface, and the interfaces between the screw threads and the surrounding bone (17). In contrast, a bonded contact condition was assumed for the interface between the cortical and cancellous bone, simulating their natural, firm attachment. For screw fixations, a preload of 50 N was applied to the screw shaft to simulate intraoperative tightening, as supported by literature (18).

All materials in the finite element model were modeled as isotropic, homogeneous, and linear elastic. The elastic modulus and Poisson's ratio for the cortical bone, cancellous bone, epiphyseal growth plate, and the titanium alloy (Ti-6Al-4V) implants were assigned based on values established in previous biomechanical literature (Table 1) (19, 20).

**Table 1:** Material properties assigned in the finite element model.

Material	Elastic Modulus (GPa)	Poisson's Ratio
Cortical Bone	12.6	0.3
Cancellous Bone	0.8	0.45
Epiphysis	0.6	0.48
Titanium Alloy	110	0.3

### 2.5 Meshing and model verification

The solid model was imported into ANSYS WORKBENCH 2021 (ANSYS, Ltd., Canonsburg, PA, USA) for meshing and finite element simulation. A local mesh refinement strategy was employed to increase the mesh density in regions of particular interest, including the screws, the bone fragment, and the screw thread regions, thereby enhancing computational accuracy in these critical areas. All model components were meshed using quadratic tetrahedral elements (SOLID187) (20). The A1 model configuration, for instance, consisted of 761,005 nodes and 468,764 elements. To ensure consistency and minimize the potential influence of mesh disparities on the comparative results, all finite element models with different fixation configurations were meshed using identical global settings.

The quality of the mesh is critical for the accuracy of finite element simulations. Therefore, a mesh convergence study was conducted prior to the formal analyses. For the initial model, the local mesh around the internal fixation devices and the epiphysis was iteratively refined. Convergence was considered achieved when the maximum displacement value varied by less than 5% across five consecutive refinement steps, with a final element size of 1 mm in these critical regions. A size-based partitioning strategy was subsequently used to generate the final mesh for the entire solid model. Following this verification process, each simulation scenario was run separately under the same boundary conditions, fixed loads, and mesh sizes, and the resulting visual and numerical outputs were recorded for analysis.

## 3 Results

The finite element analysis results demonstrated distinct biomechanical performances among the different fixation constructs. Screw fixation consistently yielded the minimal fracture fragment displacement, whereas constructs utilizing only Kirschner wires exhibited the greatest fragment displacement and structural deformation.

**Figure 4:**
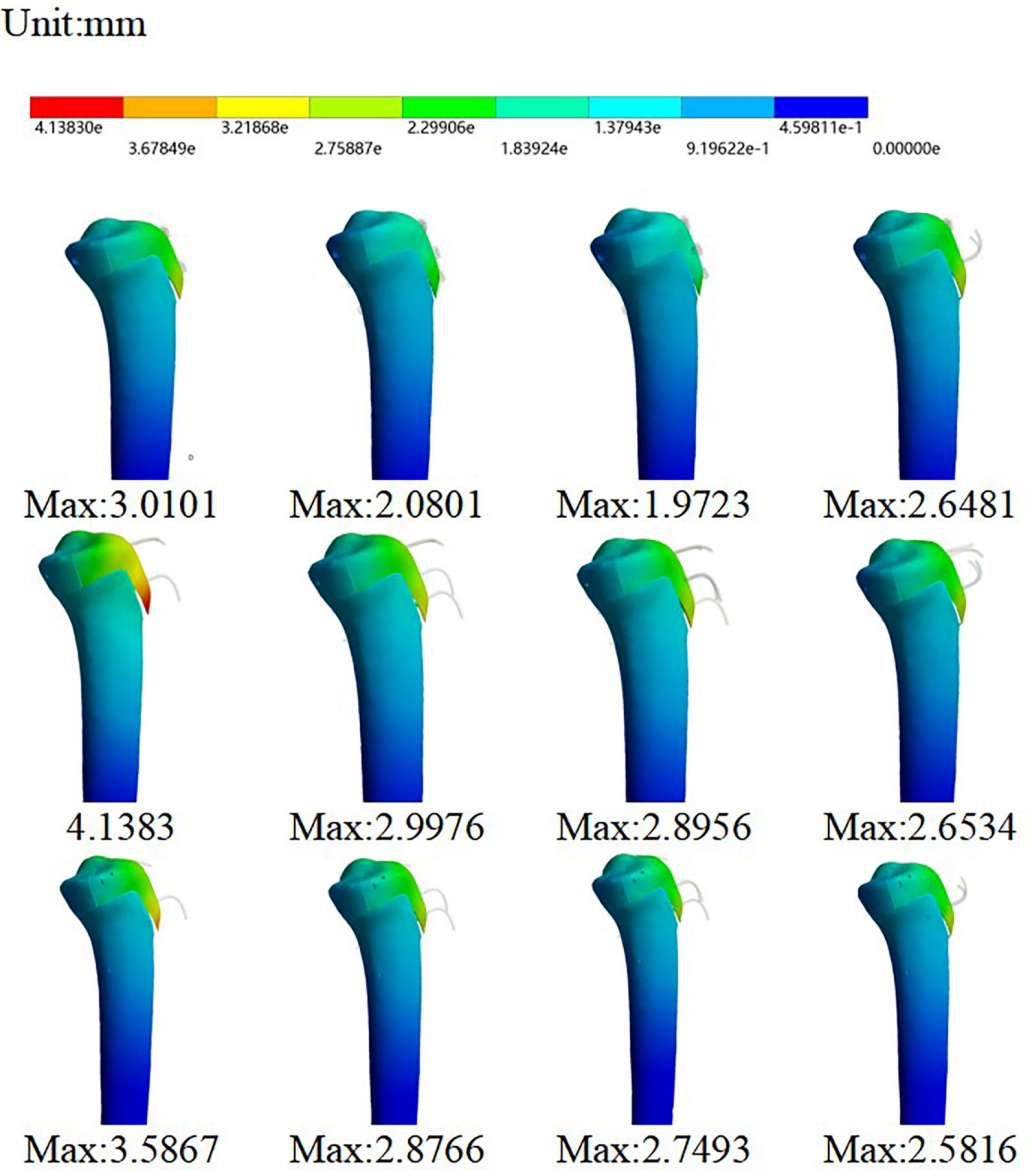
Maximum displacement of the tibial tuberosity fracture fragment for each fixation pattern.

The highest stability was observed in the C3 model (screw fixation alone), which demonstrated a fracture fragment displacement of only 1.9723 mm. This was the only configuration in which displacement remained below the 2.0 mm threshold (Figure 4). Furthermore, compared to K-wire fixations, the C3 model also produced the lowest stress values, with a bone stress of 295.79 MPa and a von Mises stress of 73.913 MPa in the internal fixation device.

**Figure 5:**
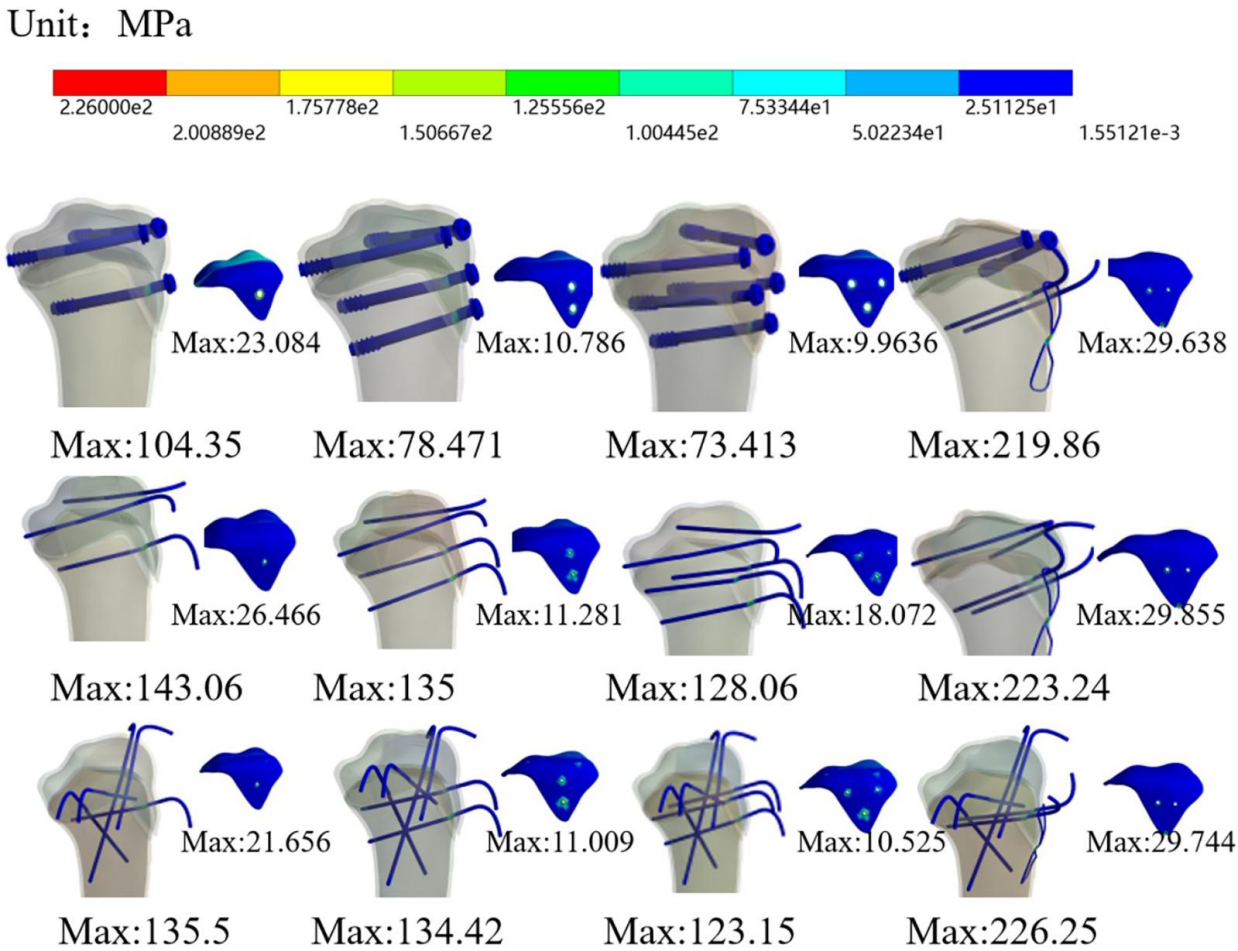
Von Mises stress distribution contour maps of the epiphysis and internal fixation.

Among the K-wire-based constructs, the C-type configuration (featuring four intersecting K-wires) demonstrated superior fixation stability compared to the B-type configuration (two non-crossing K-wires) (Figure 5). This indicates that K-wire fixation alone, regardless of the specific configuration, could not achieve a fixation strength comparable to that of screw fixation.

**Figure 6:**
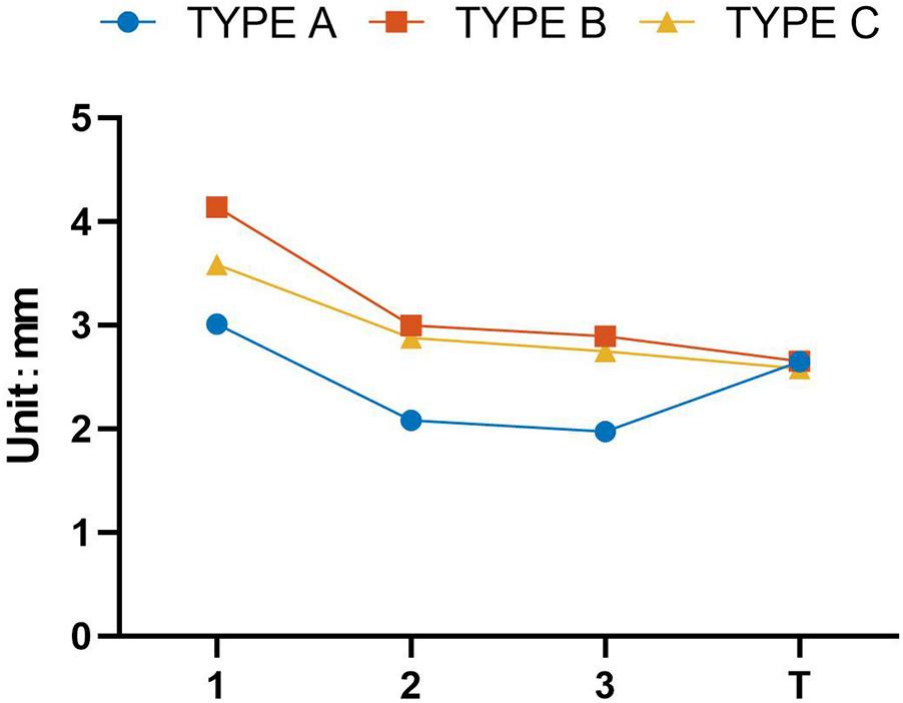
Displacement variation patterns between different models.

In the tension band fixation models (A-T, B-T, C-T), stability was similar between screw and K-wire fixation, with fragment displacement ranging from 2.58 mm to 2.64 mm. (Figure 6). However, this improvement in stability came at a cost: the tension band models were associated with significantly elevated bone stress (approximately 1,100 MPa) and induced greater epiphyseal damage compared to their non-tension-band counterparts within the same fixation group (Table 2).

**Table 2:** Bone von Mises stress of the model.

Fixed method	1	2	3	T
TYPE A (MPa)	426.40	330.74	295.79	1,102.20
TYPE B (MPa)	566.35	512.12	478.90	1,133.30
TYPE C (MPa)	925.80	614.30	463.26	1,142.90

The finite element results indicated a non-linear relationship between the number of fixators and construct stability. While increasing the number of screws or K-wires from one to two significantly enhanced fixation stability, the marginal benefit of adding a third fixator was considerably smaller.

This trend was consistent across different fixation types. In Type A (screw fixation), adding a second screw reduced fragment displacement by 0.93 mm, whereas the addition of a third screw yielded a minimal further reduction of only 0.11 mm. A similar pattern was observed in K-wire fixations. For Type B and C constructs, adding a second K-wire substantially reduced displacement by 1.14 mm and 0.71 mm, respectively. However, the introduction of a third K-wire provided only modest additional stability, reducing displacement by a mere 0.10 mm and 0.13 mm, respectively.

In summary, for both screw and K-wire fixations, the use of two implants provided dramatically superior stability compared to a single implant. In contrast, augmenting the construct with a third fixator offered only a marginal improvement in fixation strength (Figure 6).

The application of a tension band in conjunction with K-wire fixation reduced fracture fragment displacement and enhanced construct stability. Interestingly, an opposite effect was observed in screw-based fixations. Models employing a tension band with screws demonstrated inferior stability compared to the three-screw construct, though they remained superior to the two-screw configuration.

Regarding bone stress, an increase in the number of internal fixators generally led to a reduction in von Mises stress within the bone, with the screw fixation group achieving the lowest overall stress values.

## 4 Discussion

Fixation of TTAF presents a controversial issue in clinical practice, with the primary surgical goals being to minimize physeal injury and achieve superior fixation strength. Our finite element analysis provides robust biomechanical evidence supporting screw fixation as superior to K-wire fixation. A key finding from our study is that increasing the number of fixation constructs does not linearly enhance fixation stability; the stability achieved with three screws was similar to that with two screws. Furthermore, the application of tension bands elevated stress levels to approximately 1,000 MPa, a level that may be detrimental to fracture healing and poses a risk of increased physeal damage.

The incidence of Tibial Tubercle Apophyseal Fracture (TTAF) is progressively increasing, although the precise etiologies remain incompletely understood. Typical injury mechanisms involve strenuous, large-amplitude movements that precipitate acute pain, localized soft tissue swelling, and tenderness over the tibial tuberosity. Studies suggest that males are more prone to Type III fractures, while females are more susceptible to Type I fractures.This disparity could stem from greater muscle mass and elevated mechanical loading in adolescent males, which in turn results in increased pressure at the tibial tuberosity (21).

Elevated Body Mass Index (BMI) is frequently cited as a potential risk factor (2, 22, 23). However, Shin et al. (7) proposed an alternative perspective, emphasizing aberrant knee biomechanics rather than extreme BMI values. They posited that reduced bone strength in obese children due to impaired physical health, coupled with lower bone mineral density in underweight children, and amplified extensor strength related to weight, are key determinants. The work of Sheppard et al. (24) and Riccio et al. (25) further supports this viewpoint. The potential association between Osgood-Schlatter Disease (OSD) and TTAF continues to be a subject of considerable debate, with no definitive consensus yet established (2, 26). Other proposed risk factors include abrupt resumption of high-intensity activities, endocrine fluctuations (such as decreased estradiol, increased progesterone, and insulin resistance), and a history of knee pain, which may serve as a significant predictor for bilateral TTAF (27).

The unique anatomy of the developing skeleton explains the susceptibility to TTAF. From ages 9–11, a secondary ossification center appears at the tibial tuberosity. Epiphyseal closure proceeds in a posterior-to-anterior and proximal-to-distal direction, leaving the tibial tuberosity vulnerable until final fusion. Prior to fusion, the native fibrocartilage is replaced by columnar chondrocytes, which possess inferior tensile strength (28). This zone of transitional, weaker cartilage is the typical site of TTAF. Ossification begins earlier in females (approximately 10.2 years) than in males (approximately 11.8 years) (29). Recent MRI-based studies by Pennock et al. (29) detail that ossification initiates at the tip of the tuberosity epiphysis, with the proximal tibial epiphysis growing distally until fusion is achieved. Our findings on the high physeal stress induced by tension bands, compared to screws, provide a biomechanical rationale for avoiding tension band fixation in younger children with significant growth remaining, favoring K-wire fixation instead.

Surgical intervention is required in the majority of TTAF cases, with one systematic review reporting a surgical rate of 88% (28). Widely applied in clinical practice are techniques such as open reduction and internal fixation (ORIF) with compression screws, closed reduction via percutaneous K-wire fixation, fasciotomy for compartment syndrome, and arthroscopically assisted procedures. Given that the adolescent tibial tuberosity apophysis retains its growth potential, fixation strategies must account for the risk of physeal injury (14). However, excessive tension applied by sutures or tension bands increases the risk of premature physeal bridging. Nevertheless, excessive tension applied by sutures or tension bands raises the risk of early physeal closure, potentially resulting in genu recurvatum. This complication is more prevalent in skeletally immature children with tibial tuberosity avulsion fractures (TTAF) due to residual growth deformity that compromises the normal posterior tilt of the tibial plateau.

Furthermore, Pretell Mazzini et al. (30) identified symptomatic bursitis due to hardware irritation as the most frequent complication, necessitating implant removal in 53% of cases. Both the initial trauma and the surgical intervention can potentially injure the epiphysis, leading to growth disturbances, limb length discrepancy, or genu recurvatum. Fortunately, these sequelae are relatively uncommon, as TTAF typically occur near the time of physiologic physeal closure. The risk is higher in younger patients with greater growth remaining, with reported rates of genu recurvatum at 4% and limb length discrepancy at 5% in patients under 13 years of age (30, 31). Other complications, including knee stiffness, malunion, nonunion, and refracture, have been described but are generally rare.

Several limitations are acknowledged in this study. Firstly, the finite element model's reliance on the epiphyseal morphology of a single specimen prevents the consideration of inter-subject anatomical variability, potentially limiting generalizability. Secondly, the assumption of uniform, linear elastic properties for all materials and the modeling of cortical bone with a consistent 2 mm thickness may lead to an overestimation of the region's actual structural stiffness. Thirdly, while the patellar tendon force (1,654 N) was simulated as the primary load from quadriceps contraction onto the tibial tuberosity, the model did not incorporate stabilizing forces from other muscles or surrounding soft tissues, such as other tendinous units and the periosteum. Additionally, the simplified representation of the proximal tibial surface, configured solely to resist compression, restricts the accuracy of its interaction with the femoral condyles. Lastly, the modeling of fracture surfaces as perfectly smooth neglects surface irregularities inherent in real fractures, which could influence stability. Future studies are recommended to pursue more comprehensive clinical and biomechanical investigations.

## 5 Conclusion

Based on our findings, the selection of a fixation method is critical for stabilizing TTAF and mitigating complication risks, as it directly influences the load transfer to the bone fragments. This study biomechanically supports the use of screw fixation to effectively transfer applied loads to the tibia, thereby achieving high construct stability with low bone stress. The use of a tension band is not recommended due to the associated high stress concentrations on both the bone and the epiphysis. Furthermore, the application of two screws at the tibial tuberosity provides a stability comparable to that of three screws, assuming sufficient bone stock is available in the cortical fragment. The decision to employ K-wire fixation should be guided by specific surgical scenarios and patient anatomy.

## Data Availability

The raw data supporting the conclusions of this article will be made available by the authors, without undue reservation.
